# Juvenile obesity and its association with utilisation and costs of pharmaceuticals - results from the KiGGS study

**DOI:** 10.1186/1472-6963-11-340

**Published:** 2011-12-16

**Authors:** Christina M Wenig, Hildtraud Knopf, Petra Menn

**Affiliations:** 1 Ludwig-Maximilians-Universität München, Munich School of Management - Institute of Health Economics and Health Care Management, Ludwigstr. 28 RG, 80539 Munich, Germany; 2 Helmholtz Zentrum München - German Research Center for Environmental Health - Institute of Health Economics and Health Care Management, Ingolstädter Landstraße 1, 85764 Neuherberg, Germany; 3 Robert Koch Institute, Department of Epidemiology and Health Reporting, General-Pape-Str. 62-66, 12101 Berlin, Germany

## Abstract

**Background:**

According to a national reference, 15% of German children and adolescents are overweight (including obese) and 6.3% are obese. An earlier study analysed the impact of childhood overweight and obesity on different components of direct medical costs (physician, hospital and therapists). To complement the existing literature for Germany, this study aims to explore the association of body mass index (BMI) with utilisation of pharmaceuticals and related costs in German children and adolescents.

**Methods:**

Based on data from 14, 836 respondents aged 3-17 years in the German Interview and Examination Survey for Children and Adolescents (KiGGS), drug intake and associated costs were estimated using a bottom-up approach. To investigate the association of BMI with utilisation and costs, univariate analyses and multivariate generalised mixed models were conducted.

**Results:**

There was no significant difference between BMI groups regarding the probability of drug utilisation. However, the number of pharmaceuticals used was significantly higher (14%) for obese children than for normal weight children. Furthermore, there was a trend for more physician-prescribed medication in obese children and adolescents. Among children with pharmaceutical intake, estimated costs were 24% higher for obese children compared with the normal weight group.

**Conclusions:**

This is the first study to estimate excess drug costs for obesity based on a representative cross-sectional sample of the child and adolescent population in Germany. The results suggest that obese children should be classified as a priority group for prevention. This study complements the existing literature and provides important information concerning the relevance of childhood obesity as a health problem.

## Background

Obesity is one of the biggest public health problems worldwide, not only in adults but also in children and adolescents. In the WHO European region, the prevalence estimates of overweight (including obesity) in 11- to 13-year-old children range between 5% and 25% [1]. The data from the German Interview and Examination Survey for Children and Adolescents (KiGGS) show that, among 3- to 17-year-olds, 15% are overweight (including obese) and 6.3% are obese according to a national reference. Extrapolated to the German population, this leads to a total of 1.9 million overweight children, including 800, 000 who are obese [2].

Overweight and obesity in adulthood are recognised as important risk factors for numerous chronic diseases [3]. Obesity in childhood also increases the risk of later morbidities [4,5] and in turn increases the risk of obesity in adulthood [6]. Moreover, a recently published review found that childhood obesity already has an immediate impact on child health [4].

Besides the physical, mental and social health consequences of the obesity epidemic, another concern is the economic burden related to overweight and obesity. Focusing on adults, previous empirical research has demonstrated that overweight and obesity are associated with a substantial economic burden in terms of excess healthcare utilisation and productivity losses [7-10]. Evidence of the short-term excess healthcare costs associated with obesity in children and adolescents is ambiguous (for a short review of recent studies, see John et al. [11]). Although some studies have not found a positive correlation between costs and body mass index (BMI) [12,13], in other studies a positive impact was observed [14-16]. In further studies, this positive relationship was only visible in subgroups, such as adolescents [17] or girls [18]. However, comparability between these studies is difficult because of the use of different methods and the inclusion of different age groups and cost components. One study only found significant differences in prescription drug costs [15]. In a recently published article on the excess costs of overweight and obesity in German children and adolescents based on the KIGGS study [19], we found significantly higher physician costs for overweight and obese children compared with the normal weight group. However, this was not evident for therapist and hospital costs. The component of pharmaceutical utilisation and costs was not included in this previous article. The aim of this study is to fill that gap by assessing pharmaceutical utilisation and costs associated with overweight and obesity in children and adolescents in Germany.

## Methods

### Data

The data were collected in the German Interview and Examination Survey for Children and Adolescents (KiGGS), a population-based survey performed by the Robert Koch Institute [20]. From May 2003 to May 2006, a total of 17, 641 children and adolescents aged 0-17 years participated in KiGGS, yielding an overall response rate of 66.6%. The sample was derived from 167 sample points (communities) representative of the German population, stratified by federal state and community type. Within each sample point, participants were selected randomly from the official registers of local residents. Participants beyond 14 years of age and all parents provided written informed consent prior to the interview and examination. The survey was approved by the Federal Office for Data Protection and by the Charité-Universitätsmedizin Berlin ethics committee. The sampling process and study design are explicitly described in earlier articles [21-24].

According to a national reference [25], 15% of all children in the KiGGS study are classified as overweight (including obese) and 6.3% as obese. For a detailed description of the epidemiological results, see Kurth/Schaffrath-Rosario (2007) [2].

The examination took place in examination centres at the sample points. Information about sociodemographic characteristics, health and healthcare utilisation was obtained from self-administered questionnaires filled in by the parents. Data on the utilisation of pharmaceuticals during the previous 7 days were collected by a physician in a standardised computer-assisted personal interview. If participants were aged 14 years or older, they were allowed to answer these questions themselves. A detailed description of data collection and first results on medication use has been published previously [26].

Data on height and weight were obtained from physical examinations [27]. Following the recommendations from the working group on obesity in children and adolescents [28], BMI was classified into five groups according to German age- and sex-specific percentile cut-off points for children and adolescents: very underweight (< P3), underweight (P3- < P10), normal weight (P10-P90), overweight but not obese (> P90-P97), obese (> P97) [25,27].

Information on parents' income, occupational status and education was used to quantify the socioeconomic status (SES), which was categorised into low, medium and high SES [21,29-31]. Missing values for SES (n = 370, 2.7%) were imputed using the discriminant function method from the SAS procedure PROC MI based on the variables income, insurance status, migrant status, parents' BMI, utilisation of regular child health check-ups, residence (east/west Germany, urban/suburban area) and health-related quality of life (based on the KINDL^R ^total score [32,33]). Children and adolescents are defined as migrants if they emigrated from another country and at least one parent was not born in Germany or if both their parents immigrated to Germany or have no German nationality [34].

The present analyses are restricted to children and adolescents aged 3-17 years (n = 14, 836), as additional methodological problems complicate the comparison with the BMI reference values for younger children [2]. A total of 89 participants with no information on weight status were excluded from the analyses as well as another 155 participants with missing information on the utilisation of pharmaceuticals. This resulted in a population under research of 14, 592 children and adolescents.

In order to account for selection bias as far as possible, post-stratification weights were used, which have also been applied in the underlying epidemiological studies to adjust for discrepancies to the German population regarding age, sex, region and nationality. The weights also account for differences in the selection probability resulting from the sampling method, e.g. the oversampling in East Germany [23]. A detailed sociodemographic and socioeconomic description of the total study population and the subgroup of children with drug utilisation is given in Table 1. Percentage and mean values were calculated using weighted data.

**Table 1:** Sociodemographic sample description^a^

		All (N = 14, 592)		With drug utilisation (N = 5, 815)	
**Age (3.00-17.98 years)**	Mean (SE)	10.9	(0.03)	10.9	(0.06)

**Sex**	male	7, 445	(51.4%)	2, 714	(46.8%)

**BMI group**	Very underweight (< P3)	280	(1.9%)	117	(1.9%)

**(Kromeyer-Hauschild**	Underweight (P3- < P10)	752	(5.1%)	300	(5.2%)
**et al. 2001)**[25]	Normal weight	11, 357	(77.9%)	4, 491	(77.5%)
	Overweight, not obese (P90-P97)	1, 306	(8.7%)	537	(8.9%)
	Obese (> P97)	897	(6.4%)	370	(6.5%)

**Migration status**	Migrant	2, 201	(17.3%)	708	(13.9%)
	Non-migrant	12, 330	(82.7%)	5, 083	(86.1%)

**Health insurance**	Statutory insurance	12, 813	(89.1%)	5, 119	(88.6%)
	Private insurance	1, 384	(10.7%)	575	(11.3%)
	Other/no insurance	23	(0.1%)	7	(0.1%)

**Socioeconomic status (Winkler 1998) **[31]	Low SES	4, 096	(28.0%)	1, 514	(26.4%)
	Medium SES	6, 797	(45.6%)	2, 765	(46.0%)
	High SES	3, 699	(26.5%)	1, 536	(27.7%)

^a^Percentage and mean values were calculated using weighted data.

BMI, body mass index; SE, standard error; SES, socioeconomic status.

### Measurement and assessment of drug utilisation and costs

For the assessment of drug utilisation and related costs, a rather narrow definition of pharmaceuticals was applied with reference to §2 German Pharmaceuticals Act (AMG), and 'non-pharmaceuticals' were excluded based on ATC (anatomic therapeutic chemical classification) groups. Specifically, vitamins and dietary supplements (ATC A11/A12) were excluded as well as ATC groups V02-V60 (varia), homoeopathic medicines and teas. Utilisation was defined as the number of pharmaceuticals taken within the last 7 days.

Pharmaceutical costs were estimated based on information on the name and dosage of drug intake. First, the drug name was used to derive costs per package. As suggested by costing guidelines [35,36], the pharmaceuticals were priced with actual 2006 prices for the largest freely disposable package (N3) according to the national price list [37] in a conservative base analysis. If the drug name was imprecise (e.g. 'cough syrup'), the price of the most frequently mentioned pharmaceutical in the particular ATC group was used. If only the agent could be identified (e.g. 'acetylsalicylic acid/ASA'), the cheapest product in the particular ATC group was assumed.

In a second step, total drug costs per week were calculated as follows: the self-reported number of days of drug intake per week divided by the number of days a package's content was intended for (computed using the defined daily dose - DDD). This gives the proportion of the package that was used per week. This proportion was then multiplied by the package price, resulting in costs per week. The days of drug intake were documented in four categories: 'daily' and 'several times a day' were interpreted as '7 days per week'; 'often, but not daily' as '1-6 days per week' resulting in a mean of 3.5 days; and 'less often than once per week' as 'once per week' as it was mentioned in the context of medicines taken during the previous 7 days. If information on the frequency of drug intake was missing (N = 278, about 3% of all 8, 854 cases), the mean frequency in the respective ATC group was imputed instead.

In order to improve comparability with other studies, mean costs per week were extrapolated to 1 year by multiplication with a factor of 52.

To test the sensitivity of utilisation and cost estimates to changes in the underlying assumptions, univariate sensitivity analyses were performed. The number of pharmaceuticals was also analysed without excluding vitamins, dietary supplements, varia, homoeopathic medicines and teas. With regard to costs, the prices for the smallest available package size (N1) were taken instead to estimate the potential underestimation of costs due to this assumption. Furthermore, we assessed the impact of pharmacy discounts and weighting for seasonal differences in the data collection on the results.

### Statistical analysis

Mean and percentage values as well as measures of dispersion were calculated using weights to account for selection bias as far as possible [23].

In univariate analyses, the prevalence of pharmaceutical intake, in total and separated by ATC groups, was compared between BMI groups, and Chi^2 ^tests were conducted to assess the significance of differences. Moreover, the duration of intake and source (e.g. physician prescription) were analysed with regard to the five different BMI groups. The number of pharmaceuticals per child was also compared between BMI groups and tested for significance using Kruskal-Wallis tests.

In order to account for non-normality of the cost data, confidence intervals (CIs) were estimated for each BMI group applying a non-parametric bootstrap approach using a percentile method [38].

The association between BMI group and utilisation as well as costs was examined using multiple generalised mixed regression models. To analyse the relationship between BMI and the number of pharmaceuticals, we compared the performance of models using different distribution assumptions. A negative binomial model showed a better fit to the data than a Poisson model or a generalised Poisson model (based on pseudo-likelihood), so a negative binomial model was chosen. Although a large proportion of children without drug intake might be a problem, comparing the observed distribution of the number of pharmaceuticals and the distribution of the predicted values from the model indicated a satisfactory fit to the data. Also, the additional calculation of a hurdle model on drug utilisation barely affected the results regarding BMI.

We report the exponents of regression estimates that can be interpreted as factors.

To allow for the typically skewed distribution of costs and the high proportion of participants without costs, a two-step model was applied [39]. As a first step, the association of BMI and the probability of drug intake was analysed. Reducing the analysis to cases with drug intake and therefore positive costs, in a second step, the association of BMI and total costs was analysed using a gamma model with log-link function [40,41]. The modified Park test supported the choice of the Gamma distribution (p = 0.58), and the Hosmer-Lemeshow-Test (p = 0.90), the Pregibon Link-Test (p = 0.80) as well as the Pearson Correlation Test (p = 0.58) all confirmed the choice of the log link function.

All regression analyses were adjusted for the variables sex, age, SES (low, medium or high), as well as migrant status. These variables were shown to have an important impact on the utilisation of pharmaceuticals in a previous study [26]. To allow for a possible non-linear relationship, a quadratic term for age was added. As data collection was slightly more frequent in the autumn (29.7%) and winter (24.6%) than in the spring (22.4%) and summer (23.2%), we additionally adjusted our analyses for seasonal effects to avoid possible confounding, even though seasonal differences between BMI groups were not significant overall. The sample point was included as a random effect, and sample weights were used to account for the complex sample design.

To show the importance of pharmaceutical costs in children and adolescents, mean annual pharmaceutical costs were added to further cost components (hospital stays, physician and therapist visits), that were reported in an earlier article [19]. For this, we used a subsample of 14, 075 participants where information on both, pharmaceutical and non-pharmaceutical utilisation was available. 95% confidence intervals were again estimated based on a non-parametric bootstrap approach.

Statistical analyses were performed using the software SAS (SAS Institute Inc., Cary, NC, USA, version 9.2), and the significance level for all analyses was 5%.

## Results

### Utilisation of pharmaceuticals by BMI groups

The mean prevalence of drug intake is slightly higher among overweight and obese children (both 40.9%) compared with normal weight (39.7%) children and adolescents (overall Chi^2 ^test for differences between BMI groups not significant). These differences are more distinct in the ATC groups G (genito-urinary system and sex hormones), M (musculo-skeletal system) and N (nervous system). The mean number of drugs also increases slightly from normal weight (0.59) to obese (0.61) children. However, the overall Kruskal-Wallis test for differences between BMI groups was not significant. This increase was observed especially in the ATC groups G (genito-urinary system and sex hormones), H (systemic hormonal preparations, excluding sex hormones and insulins), J (antiinfectives for systemic use), M (musculo-skeletal system) and N (nervous system). Further analyses of utilisation patterns shows that obese children have a higher percentage of drugs prescribed by a physician (p = 0.04) and a somewhat (but not significantly) higher percentage of long-term (> 1 year) medication (p = 0.15) compared with normal weight children.

Regarding the results of the regression model, BMI, sex, age, SES and migrant status are significantly associated with the number of pharmaceuticals taken during 1 week: overweight and obese children take more drugs than normal weight children (difference significant only for obese children), and girls take more pharmaceuticals than boys. The effect of age is U-shaped. Moreover, drug utilisation is higher for children with medium and high SES and a non-migrant background. Comparing these results with a model including all the pharmaceuticals that were mentioned (before limiting the pharmaceuticals definition by excluding vitamins, etc.) reveals that the effect of BMI becomes stronger, whereas the effect of SES is less important after the exclusion. The results of the two regression models are summarised in Table 2.

**Table 2:** Number of pharmaceuticals - results of regression analysis

	Number of drugs (after exclusion)		Number of drugs (before exclusion)	
**Parameter**	**Exp(Est)**	**Pr > |t|**^**e**^	**Exp(Est)**	**Pr > |t|**^**e**^

Intercept	1.472	< 0.0001	1.608	< 0.0001

Sex: female	1.225	< 0.0001	1.145	< 0.0001

Age	0.796	< 0.0001	0.820	< 0.0001

Age squared	1.011	< 0.0001	1.009	< 0.0001

BMI^a^: very underweight	1.080	0.0332	1.160	0.0891
underweight	1.070		1.091	
overweight	1.077		1.035	
obese	1.140**		1.060	

Socioeconomic status^b^: high	1.099**	0.0154	1.234***	< 0.0001
medium	1.075*		1.121***	

Season^c^: spring	0.944	0.1102	0.935	0.0178
summer	0.270*		0.848**	
autumn	0.951		0.955	

Migrant^d^	0.773***	< 0.0001	0.739***	< 0.0001

N = 14, 531.

Negative binomial model, random effect: sample point, dependent variable: number of pharmaceuticals.

Reference: ^a^normal weight; ^b^low socioeconomic status; ^c^winter; ^d^non-migrant.

^e^p-value of total effect.

Significance levels for individual effect levels: *** < 0.001, ** < 0.01, * < 0.05.

BMI, body mass index.

### Costs by BMI groups

Table 3 shows mean costs (per week and per year) by BMI group. The relationship between BMI and drug costs is U-shaped with a minimum in the normal weight group.

**Table 3:** Drug costs (in €) by BMI groups

Weighted means N = 14, 592	Mean pharmaceutical costs/week		Mean pharmaceutical costs/year	
	**Mean**	**95% CI**^**a**^	**Mean**	**95% CI**^**a**^

**Very underweight**	7.54	[3.04-16.87]	392	[158-877]
**Underweight**	4.54	[3.49-5.82]	236	[181-303]
**Normal weight**	3.27	[3.03-3.54]	170	[158-184]
**Overweight**	3.31	[2.75-3.95]	172	[143-205]
**Obese**	4.06	[2.98-5.60]	211	[155-291]

**Total**	**3.47**	**[3.22-3.76]**	**181**	**[167-195]**

^a^Confidence intervals (CIs) were estimated based on 5, 000 bootstrap replications.

The results of the two-step regression analysis of pharmaceutical costs (Table 4) suggest that the likelihood of drug intake does not differ significantly between BMI groups (step 1) but, considering only those children who took a medicine (step 2), there are BMI related differences in the amount of costs: compared with normal weight children, costs for obese children are 24% higher. In addition, the positive estimate for higher SES is only significant in the second step of the model. Girls have a higher probability of incurring costs than boys but, if they take a drug, the mean costs are lower. Age shows a significant association with the probability of incurring costs, but not with the total costs in the second step. Children with a migrant background have a lower probability of incurring costs at all but, if they do, costs are higher than in the non-migrant group.

**Table 4:** Pharmaceutical costs - results of two-step regression analysis

	**1. Probability**^**a**^ N = 14, 531		**2. Amount of costs**^**b**^ N = 5, 791	
**Parameter**	**Odds ratio**	**Pr > Chi**^**2g**^	**Exp(est)**	**Pr > |t|**^**g**^

Intercept	-	-	9.5571	< 0.0001

Sex: female	1.351	< 0.0001	0.849	< 0.0001

Age	0.728	< 0.0001	0.970	0.1559
Age squared	1.015	< 0.0001	1.000	0.6812

BMI^c^: very underweight	1.006	0.3100	2.461***	< 0.0001
underweight	1.077		1.387***	
overweight	1.129		1.049	
obese	1.122		1.237**	

Socioeconomic status^d^: high	1.075	0.4349	1.144**	0.0283
medium	1.034		1.069	

Season^e^: spring	0.960	0.3520	0.973	0.7188
summer	0.869		1.152	
autumn	0.955		0.900	

Migrant^f^	0.673***	< 0.0001	1.144*	0.0144

^a^Logistic mixed regression; ^b^Generalised linear mixed regression model (Gamma distribution with log-link);

Reference: ^c^normal weight, ^d^low socioeconomic status, ^e^winter, ^r^non-migrant.

^g^p-value of total effect.

Significance levels for individual effect levels: *** < 0.001, ** < 0.01, * < 0.05.

BMI, body mass index.

Separate analyses for age groups (3-6, 7-10, 11-13, 14-17 years) show no significant differences for overweight and obese children regarding the probability of incurring costs (step 1) in all age groups, and significantly higher costs for the obese regarding total costs (step 2) in all age groups except in the 11- to 13-year-olds.

### Sensitivity analysis

To assess the sensitivity of cost results to changes in the assumptions, alternative approaches concerning seasonal differences, pharmacy discounts and package size were applied. Accounting for seasonal differences in data collection by weights led to an increase in total costs of 0.6%. The inclusion of pharmacy discounts resulted in costs decreasing by 8.7%. The strongest effect was visible for the alternative assumption concerning package sizes: taking the smallest package available (usually N1) as a basis for cost estimation would lead to an increase in costs of 21.5%. The estimated costs would be 20% lower if the analysis only included physician-prescribed drugs.

**Figure 1:**
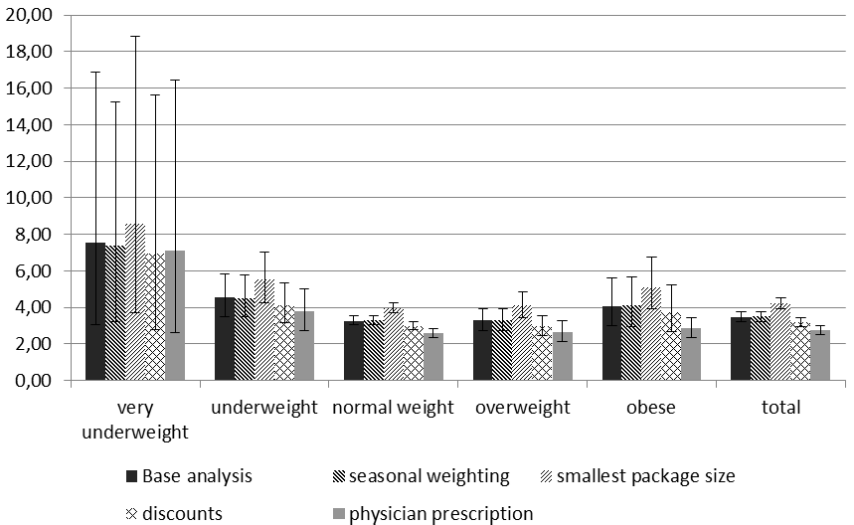
**Sensitivity analyses (mean costs per week in €)**.

However, although all these changes affected the total costs, the differences between BMI groups remained similar. Figure 1 illustrates the results of sensitivity analyses in total and by BMI group.

### Total costs per year

**Figure 2:**
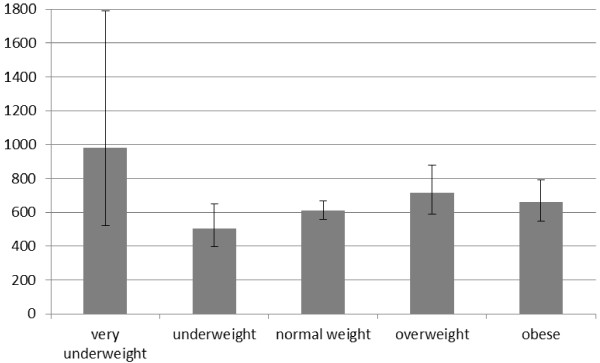
**Mean total annual costs (in €)**.

In a previously published article mean costs for hospital stays as well as physician and therapist visits per year were reported as € 442 [19]. Based on this study, pharmaceutical costs would add another 41% to that amount, resulting in total mean annual costs of € 623 (95% CI [579-671]). Figure 2 displays the mean annual total costs for the five BMI-groups. In total, mean annual costs were significantly higher for overweight and obese participants compared to the normal weight group (p < 0.0001).

## Discussion

The primary aim of this study was to explore the association between BMI and the utilisation of pharmaceuticals and costs in German children and adolescents. There is no significant correlation between BMI group and the probability of drug utilisation. However, the number of pharmaceuticals used is 14% higher for obese children compared with normal weight children. Furthermore, there is a positive trend to physician-prescribed medication in obese children and adolescents. Regarding those children with drug use, costs are 24% higher for obese children compared with the normal weight group. Thus, a positive association of childhood obesity and drug utilisation and costs is already visible in children and adolescents. A comparison with physician, therapist and hospital costs shows that pharmaceutical costs make an important component of total healthcare costs in children and adolescents.

This is the first study estimating the excess drug costs resulting from obesity based on a representative cross-sectional sample of the German child and adolescent population using a bottom-up approach. One of the main advantages of this approach is the possibility of comparing utilisation and costs in population subgroups, for example with respect to sociodemographic variables and BMI. Although analyses based on comprehensive administrative statistics might give better estimates of the actual level of expenditure for the respective institutions, they are mostly not a representative sample of the population and do not include patients' out-of-pocket expenditures. However, in the German healthcare system, out-of-pocket expenditures are especially relevant for pharmaceuticals. Furthermore, these studies often do not include clinical data, such as measured weight for height, and are therefore limited to cases of diagnosed obesity.

However, several limitations of this study must be pointed out. Most importantly, analyses were based on a cross-sectional survey. Therefore, the results allow for conclusions concerning correlations, but not causal relationships. While obesity was shown to increase the risk of numerous health problems, some illnesses might also induce weight gain. This sample included underweight children and adolescents, although these were not the focus of our analyses. The results show that very underweight children and adolescents cause the highest mean costs, but also had the highest standard deviation. Though underweight is not associated with the probability of incurring costs, there is a significantly positive association with total costs. However, on account of the relatively small percentage of cases, the results for this group should be interpreted with caution. High pharmaceutical costs resulting from low weight seem plausible as extreme underweight might lead to an impaired immune system [42]. However, causality is again unclear, as extreme underweight might also be a consequence of severe or enduring illness, which itself implies increased healthcare utilisation. To definitely answer the question of causality, longitudinal data are required.

Although the problem of recall error should be small considering the short time period, it cannot be excluded as participants are asked to provide information retrospectively - in this case, to state the utilisation of pharmaceuticals for the previous 7 days. Moreover, the results may not be fully representative of the total population with regard to sociodemographic characteristics. These cross-regional differences were reduced by post-stratification weighting for age, sex, region and nationality [23]. Furthermore, as about 34% of those contacted did not respond to the survey, non-response bias cannot be excluded. Costs may be underestimated, because very sick children might not have taken part in the study. However, extensive non-response analyses have been conducted that show only moderate differences in sociodemographic as well as health-related characteristics [23]. As far as non-response is explained by age, sex, region or nationality, it is accounted for by using the respective weights. Statistical analyses did not include adjustments for comorbidities. Correlation between health problems and overweight is not trivial; thus, the excess cost approach tries to capture all the differences between the analysed BMI groups.

Regarding the estimation of drug costs, several assumptions were necessary that may have caused over- or underestimation of costs. The estimation of pharmaceutical costs was based on the DDD, as suggested by the WHO. However, this measure tends to overestimate drug consumption: first, if pharmaceuticals are not specifically for children, the DDD refers to the daily dose for adults; second, it presumes full compliance. Furthermore, the frequency of drug intake was estimated based on the four response categories 'daily' and 'several times a day', 'often, but not daily' and 'less often than once per week'. It is not clear how this affects cost results. Yet, data for a more precise population-based assessment of pharmaceutical costs are not available so far.

Sensitivity analyses were performed regarding package sizes, seasonal effects and legal price discounts. Discount contracts between the pharmaceutical industry and healthcare insurers could not be taken into account, because they are not publicly available. Although all these changes affected the extent of costs in total, none affected the differences between BMI groups.

In this study, we estimated the costs for drug consumption, not actual expenditures, which might be even higher, if packages are only partly used and leftovers are thrown away. As utilisation of pharmaceuticals was requested for the last 7 days, the extrapolated yearly cost estimates should be interpreted with caution.

SES (based on parental income and education) was included as a confounder in statistical analyses because it may influence health care utilisation as an 'enabling factor' [43]. Yet, it has to be noted that SES may also be associated with overweight and obesity, but the direction of the causal relationship is not clear [44,45]: Although low income might have a negative impact on health behaviour resulting in weight gain, overweight and obesity in adults could also impede labour market outcomes and cause lower wages [46] - in our case this is only relevant if we assume a high correlation of the weight status of parents and their children. However, a recalculation of the regression model without SES as a confounding variable did not change our results.

The medical literature often questions BMI as a valid and accurate measure of overweight and obesity [47]. Especially for younger children, alternative approaches have been proposed with a slightly higher sensitivity [48]. However, the information required to compute the BMI is easy to collect and common in a number of social science data sets. A recently published study suggests that BMI serves as a good surrogate marker for obesity in population studies [49].

## Conclusion

This study complements the existing literature and provides new country-specific evidence on the relevance of childhood overweight and obesity as a health problem in Germany. A positive association of obesity and drug utilisation and costs is already visible in children and adolescents. Thus, our results suggest that obese children should be classified as a priority group for prevention. Prevention programmes with sustainable positive medical effects have a high likelihood of being evaluated as cost-effective. Yet, further research on the long-term relationship between obesity and related healthcare utilisation and costs is essential to answer the question of causality and to improve the evidence needed for economic evaluations.

## Competing interests

The authors declare that they have no competing interests.

## Authors' contributions

CW and PM developed the design and analysis plan of this study and performed statistical analyses. CW drafted the manuscript. HK was involved in the coordination/conception of the KiGGS-study. All authors (CW, HK, PM) contributed to interpretation of findings, critically reviewed each draft of the manuscript, contributed to writing and approved the final manuscript.

## Pre-publication history

The pre-publication history for this paper can be accessed here:

http://www.biomedcentral.com/1472-6963/11/340/prepub
